# Cross-sectional relationship between physical fitness components and functional performance in older persons living in long-term care facilities

**DOI:** 10.1186/1471-2318-6-4

**Published:** 2006-02-07

**Authors:** Amika S Singh, Marijke JM Chin A Paw, Ruud J Bosscher, Willem van Mechelen

**Affiliations:** 1 VU University Medical Center, EMGO-Institute, Department of Public Health van der Boechorststraat 7, 1081 BT Amsterdam, The Netherlands; 2 Vrije Universiteit, Faculty of Human Movement Sciences, van der Boechorststraat 9, Amsterdam, The Netherlands

## Abstract

**Background:**

The age-related deterioration of physiological capacities such as muscle strength and balance is associated with increased dependence. Understanding the contribution of physical fitness components to functional performance facilitates the development of adequate exercise interventions aiming at preservation of function and independence of older people. The aim of the study was to investigate the relationship between physical fitness components and functional performance in older people living in long-term care facilities.

**Methods:**

*Design *cross-sectional study

*Subjects *226 persons living in long-term care facilities (mean age: 81.6 ± 5.6).

*Outcome measures *Physical fitness and functional performance were measured by performance-based tests.

**Results:**

Knee and elbow extension strength were significantly higher in men (difference = 44.5 and 50.0 N, respectively), whereas women were more flexible (difference sit & reach test = 7.2 cm). Functional performance was not significantly different between the genders. In men, motor coordination (eye-hand coordination) and measures of strength were the main contributors to functional performance, whereas in women flexibility (sit and reach test) and motor coordination (tandem stance and eye-hand coordination) played a major role.

**Conclusion:**

The results of this study show that besides muscle strength, fitness components such as coordination and flexibility are associated with functional performance of older people living in long-term care facilities.

This suggests that men and women living in long-term care facilities, differ considerably concerning the fitness factors contributing to functional performance. Women and men may, therefore, need exercise programs emphasizing different fitness aspects in order to improve functional performance.

## Background

Ageing is associated with a deterioration of various physiological capacities, such as muscle strength, aerobic capacity, neuro-motor coordination, and flexibility. These age-related declines result in a host of negative outcomes, including functional limitations [1] and therefore loss of independence. Due to their frailty, persons living in long-term care facilities are at increased risk of further decline. Physical functioning, or the ability to perform activities of daily living, essentially contributes to the quality of life of older persons [2-4]. In order to preserve physical functioning, and thus quality of life, it is of particular importance to understand the extent to which muscle strength, motor coordination, and flexibility contribute to functional performance. In general this relationship is insufficiently understood. Scientific evidence supports that several physical fitness components are modifiable even in older people [5,6]. If these modifiable factors, contributing significantly to functional performance, can be identified, appropriate exercise programs can be tailored to the specific needs of the elderly in order to preserve physical function and independence [7].

Several studies have demonstrated a positive association between muscle strength of the lower extremities and mobility tasks such as rising from a chair [8-10] and walking speed [8,11-13]. The relationship between upper extremity strength and functional performance is less clear. Among community-dwelling elderly only weak cross-sectional correlations were found between self-reported functional status and single upper extremity strength measures, i.e. chest press strength and upper back power [14]. Cress et al. [15] investigated the relationship between muscle strength of the upper extremities and several functional performance tasks and found a significant positive relation. The relationship between other fitness components and functional performance is studied to a far lesser extent, even though balance was identified as a contributor to mobility tasks by some investigators [16,17].

Functional performance requires the complex interaction of physiologic, psychological, social, environmental and health-related factors [18]. In this paper we focus on physiological factors. Assuming that functional performance is the sum of many fitness components [16,19], this paper aims at identifying essential correlates of functional performance. Therefore, the relationship between a variety of physical fitness components and functional performance among inhabitants of long-term care facilities was investigated.

## Methods

### Subjects

Participants of the study were living in six long-term care facilities in the North-Western part of The Netherlands. Subjects were recruited to participate in a randomised controlled trial, examining the effects of three different exercise protocols on physical functioning, psychological well being, and medical consumption [20]. In each home all residents were invited to informative meetings organized in the homes. At these informative meetings the study was explained in detail. At the end of the meeting, subjects received a form on which they could assert whether they were interested in the study.

Subjects had to meet the following inclusion criteria to participate in the study: (1) aged 65 or older, (2) living in a nursing home or residential care facility, (3) able to walk six meters or more (with or without a walking aid), (4) able to comprehend the study procedures, (5) no medical contraindication for study participation, (6) no terminal disease or progressive deterioration of health, (7) and not moving away from the home within the six months intervention period (5 and 6 were evaluated by their general practitioner). Two questions from the Mini-Mental State Examination (MMSE) were used to evaluate the subjects' ability to follow an easy command [21]: (1) read the words on this card and then carry out the order: 'close your eyes', (2) I am going to give you a piece of paper, take this paper in your right hand, fold the paper in half with both hands, and put the paper down on your lap'.

Informed consent was obtained from all subjects. The study was approved by the medical ethical committee of the VU University Medical Center.

For the present analysis only baseline measurements were used. Baseline data of physical fitness and functional performance were collected in 226 participants.

### Measurements

#### General characteristics

Information on demographic and lifestyle characteristics was obtained in a personal interview. Perceived health and quality of life were rated by two single questions 'How would you rate you health?' and 'How would you rate your quality of life?' on a five-point scale. The level of physical activity was estimated by the validated Lasa Physical Activity Questionnaire [22], addressing the following activities: walking outdoors, bicycling, light household activities, heavy household activities, and a maximum of two sports activities. Respondents were asked how often and for how long in the previous two weeks they had engaged in each activity. From the questionnaire data, activity in min/d was calculated by multiplying the frequency and duration of each separate activity in the previous two weeks and dividing the multiplied score by 14. A total activity score was calculated by summing all separate activity scores.

#### Anthropometry

Body height was measured to the nearest 0.5 cm with the subject in standing position wearing no shoes, using a wall-mounted stadiometer. Body weight and body composition measurements (lean body mass, percentage body fat) were performed with Dual-Energy X-ray Absorptiometry (DEXA) (HOLOGIC QDR-2000, Hologic Inc., Massachusetts, USA). Body Mass Index (BMI) was calculated as body weight in kilograms divided by the squared height in meters.

#### Physical fitness and functional performance

Two trained research assistants measured physical fitness and functional performance according to a standardized protocol. Table 1 contains a short description of the tests measuring functional performance and those measuring physical fitness, grouped in three categories: strength, flexibility, and coordination.

**Table 1:** Brief description of the functional performance and physical fitness tests.

	Description	Score
Functional Performance tests		
chair rise	Time to rise from a straight-backed chair five times as fast as possible, if possible with the arms folded across the chest [38].	time (s)
walking eight meters	Time to walk 8 meters as fast as possible with or without usual walking aid [39]. Best of two trials [39].	time (s)
putting a coat on and off	Time needed to put a lab coat on and off [40].	time (s)
picking up a pen	Time needed to pick up a pen from the floor while standing [41].	time (s)
Physical Fitness Tests		
Muscle strength	Measured by hand held dynamometers (Micro-Fet2, Hoggan Health Industries Inc., Almere, The Netherlands). Best of three trials.	
knee extension	The subject is seated, extending the knee against the resistance of the examiner.	N/m
dorsal flexion ankle	The subject is lying in supine position, flexing the ankle against the resistance of the examiner.	N/m
elbow extension	The subject is lying in supine position, extending the elbow against the resistance of the examiner.	N/m
Coordination		
one leg stance	Time a subject is able to stand on one leg [41]. Best of two trials.	time (s)
tandem stance	Time a subject is able to stand on two legs, with the heel of one foot directly in front of and touching the toes of the other foot, with eyes opened [41,42]. Best of two trials.	time (s)
eye-hand-coordination	Time to transfer 40 blocks from a full board to an empty board in a prescribed sequence as quickly as possible with the preferred hand [23,43,44].	time (s)
reaction time	Time to react to the onset of a light by pushing a button as fast as possible. Median of 15 trials [23].	time (s)
Flexibility shoulder circumduction	Best of three trials. The subject is instructed to bring a rope, with a fixed and a movable handle, symmetrically over the head and behind the body while keeping the arms extended and the hands as close together as possible [23].	cm
sit and reach	The subject is instructed to sit on the floor, legs outstretched, in front of a standard 'sit-and-reach' box. The subject has to push bending forward [23].	cm

Physical fitness was assessed using four components of the validated Groningen Fitness Test for the Elderly (GFE): manual dexterity (coordination), reaction time (speed of movement), and flexibility of the hip and spine, and of the shoulder [23]. The Groningen Fitness test for Elderly is a reliable and valid test for the evaluation of physical fitness in the elderly (Intra Class Correlation coefficients (ICC) of individual tests varying from .83 to .96) [23]. Standing balance was assessed by the tandem stance and one-leg stance (ICC's varying from .70 to .90) [24,25]. Muscle strength was assessed by means of hand held dynamometry, which has shown to be valid and reliable in comparable study populations (ICC's and Pearson's correlation coefficients varying from .85 to .95) [26-28]. Because of the high respondent burden of tests measuring submaximal exercise capacity and maximal aerobic power, these components were not included. Furthermore, four performance tests were conducted in which participants were asked to perform daily activities in a standardized manner. We chose for performance tests that are acceptable to the participants, feasible for use in different long-term care facilities, and that equipment required for testing is inexpensive and easily transportable. Detailed descriptions of the reliability and validity of the performance tests are described elsewhere [25,26,29-32]. In brief, all tests have acceptable test-retest reliability and appropriate concurrent validity with self-reported measures of physical function.

### Statistics/data analysis

All data were analysed using SPSS for Windows (release 10.1.4).

Subjects who could not perform a test without aid or did not perform the test properly were given the worst possible score.

Differences in means were examined using Mann-Whitney *U *tests. By means of the Spearman correlation coefficient (r_s_) the univariate relationship between functional performance and physical fitness was examined.

Factor analysis was performed in order to create a summary Functional Performance Score (FPS = chair rise, picking up a pen, putting a cardigan on and off, walking). Factor analysis is often used in data reduction, for example to identify one factor (FPS = dependent variable) that explains most of the variance observed in a larger number of independent variables (chair rise, picking up a pen, putting a cardigan on and off, walking = independent variables). We chose to exclude cases that had missing values in any of the variables used for the factor analysis (missing listwise option).

To establish the nature of the relationship between functional performance and physical fitness, regression models (stepwise forward method) were fit to the data, with body weight as a covariate. Analyses were conducted for men and women separately. Values of p lower than 0.05 were considered to indicate statistical significance.

## Results

### General characteristics

General and anthropometric characteristics of the subjects are presented in table 2. Eighty-one percent of all participants was female, 19% was male. The mean age of all participants was 82 (± 5.6). The mean percentage body fat was 44% (± 7.6) and 32% (± 7.6), in women and men, respectively. Forty percent of the participants perceived their health as excellent or good, 48% as reasonable and 12% as variable or bad. More than half of the subjects (58%) considered their quality of life as reasonable, 26% judged it as excellent or good and 16% as variable or bad.

**Table 2:** General characteristics of the participants.

Variable (*n = 226*)	male	female
sex (percentage)	19 (n = 42)	81 (n = 184)
mean age (± sd)	81.6 (± 4.5)	81.5 (± 5.8)
mean percentage fat (± sd)	32.7 (± 8.7)	43.2 (± 7.9)
mean BMI (± sd)	26.8 (± 4.3)	29.1 (± 5.3)
marital status (percentage)		
- *not married*	0.0	4.9
- *married*	50.0	14.7
- *widowed*	45.2	78.1
- *divorced*	4.8	2.2
type of residence (percentage)		
- *residential care*	16.7	17.0
- *extended care*	82.3	83.0
Perceived health (percentage)		
- *excellent/good*	40.5	39.9
- *reasonable*	50.0	47.6
- *variable/bad*	9.5	12.5
quality of life (percentage)		
- *excellent/good*	28.6	25.7
- *reasonable*	59.5	57.9
- *variable/bad*	11.9	16.4
level of physical activity (minutes per day, median)	94.3	105.0
medication use (% no medication used during the last 6 months)	29.9	25.0

### Differences in physical fitness and functional performance between men and women

Results of the physical fitness and functional performance tests for men and women are shown in table 3. The number of subjects who were able to perform the one leg stance was the lowest: only 166 subjects (74%). The tandem stance test and the shoulder circumduction test were also completed by a smaller number of participants: 172 (76%) and 176 participants (78%), respectively.

**Table 3:** Functional performance and fitness tests.

Test (unit)	number able to perform test (%)	median (10^TH ^– 90^TH ^percentile)	number able to perform test (%)	median (10^TH ^– 90^TH ^percentile)	p-value † (test for difference between male and female subjects)
	*male subjects (n = 42)*		*female subjects (n = 184)*		
functional tests					
chair rise performance (s)	32 (76.2)	24,4 (15,3 – 63,6)	158 (85.9)	23,1 (16,0 – 63,6)	.80
put on a coat (s)	39 (92.9)	20,1 (11,2 – 50,1)	172 (93.5)	17,6 (11,0 – 43,3)	.33
pick up a pen (s)	36 (85.7)	3,3 (1,9 – 18,4)	163 (88.6)	3,3 (2,0 – 18,4)	.61
walking velocity (m/s)	36 (85.7)	1,2 (,0 – 1,6)	163 (88.6)	1,0 (,0 – 1,4)	.13
fitness tests					
STRENGTH					
knee extension (N/m)	41 (97.6)	113,5 (57,6 – 185,5)	180 (97.8)	69,0 (31,5 – 133,0)	.00
elbow extension (N/m)	41 (97.6)	128,0 (63,2 – 193,3)	181 (98.4)	78,0 (45,6 – 142,0)	.00
ankle dorsiflexion (N/m)	41 (97.6)	89,0 (42,0 – 152,6)	184 (100)	77,0 (47,5 – 141,0)	.20
COORDINATION					
reaction time (s)	42 (100)	246,0 (203,6 – 334,0)	182 (98.9)	262,5 (209,0 – 376,5)	.08
eye-hand-coordination (s)	42 (100)	64,6 (50,3 – 88,7)	180 (97.8)	61,5 (50,5 – 89,9)	.49
tandem stance (s)	35 (83.3)	8,6 (,0 – 30,0)	137 (74.5)	6,1 (,0 – 30,0)	.08
one leg stance (s)	34 (81.0)	2,9 (,0 – 16,9)	132 (71.7)	2,6 (,0 – 17,9)	.40
FLEXIBILITY					
sit and reach (cm)	30 (71.4)	11,3 (,0 – 27,6)	152 (82.6)	18,5 (,0 – 31,3)	.00
shoulder circumduction (cm)	35 (83.3)	51,3 (44,3 – 54,6)	141 (76.6)	52,4 (44,3 – 56,7)	.06

† = Difference between groups tested with Mann-Whitney *U *test.

Regarding functional performance, men and women did not significantly differ. Men were significantly stronger with respect to knee extension strength (difference = 44.5 N/m) and elbow extension strength (difference = 50.0 N/m), whereas women were significantly more flexible (difference sit and reach test = 7.2 cm) than their male counterparts. Because of these gender differences, the following analyses were performed for male and female subjects separately.

For both, male and female participants, investigation of the data show ceiling effects for the balance test (tandem stance).

### Bivariate correlations between functional performances and physical fitness measures

Table 4 presents Spearman correlation coefficients between all physical fitness tests, functional performance tests, and the FPS, for men and women separately.

**Table 4:** Spearman correlations between physical fitness and functional performance for men and women.

*men (n = 42)*		chair rise	pick up a pen	put on a coat	walking velocity	Functional Performance Score
muscle strength	knee extension	-.27	-.07	-.42**	.12	-.21
	elbow extension	-.31*	.03	-.48 **	.16	-.26
	ankle dorsiflexion	-.43**	-.50**	-.44**	.63**	-.63**
coordination	one leg stance	-.15	-.21	-.27	.44**	-.33*
	two leg stance	-.24	-.25	-.24	.49**	-.41**
	eye hand coordination	.64**	.43**	.54**	-.60**	.69**
	reaction time	.39*	.42**	.10	-.53**	.47**
flexibility	sit and reach	-.14	-.32*	-.42**	.40**	-.39*
	shoulder circumduction	-.15	-.08	-.43**	.14	-.22

*women (n = 184)*						
muscle strength	knee extension	-.18*	-.08	-.16*	.18*	-.18*
	elbow extension	-.17*	-.02	-.13	.18*	-.15*
	ankle dorsiflexion	-.24**	-.08	-.26**	.31**	-.26**
coordination	one leg stance	-.17**	-.23**	-.24**	.29**	-.28**
	two leg stance	-.32**	-.28**	-.25**	.43**	-.40**
	eye hand coordination	.18*	.33**	.40**	-.32**	.37**
	reaction time	.18*	.33**	.25**	-.24**	.30**
flexibility	sit and reach	-.38**	-.44**	-.39**	.42**	-.49**
	shoulder circumduction	-.33**	-.23**	-.47**	.28**	-.39**

* p < 0.05, ** p < 0.01

In men, walking velocity was highly correlated with ankle dorsiflexion strength (r = .65) and picking up a pen (r = .66). Chair rise performance was also highly related to picking up a pen (r = .72). The tandem stance (r = -.45), eye-hand coordination (r = .62), and ankle dorsiflexion strength (r = -.63) were the physical fitness tests that showed the strongest correlations with the FPS. For women, walking velocity was highly correlated with chair rise performance (r = -.61) and picking up a pen (r = -.61). The one leg stance (r = -.45), tandem stance (r = -.46), and the sit and reach (r = .45) tests showed the strongest correlations with the FPS.

### Physical fitness components as correlates of functional performance

Table 5 presents the results of the regression analyses, including F-values, standardized beta's and the explained variance that can be accounted for in the various models. For men, coordination and strength explained most of the variance of functional performance. The proportion of variance explained by the regression models for the FPS and the individual performance tests in male subjects varied between 22% and 54%. For women, flexibility (sit and reach test) and coordination (tandem stance, eye-hand coordination, and reaction time) were the most important correlates of functional performance. The proportion of variance explained by the regression models for functional performance in women varied between 19% and 37%. In women, fitness components from all domains (coordination, flexibility, and strength) predicted the FPS, whereas in men, only fitness components from the coordination domain predicted the FPS.

**Table 5:** Contribution of the individual fitness tests to the functional performance tests and the total Functional Performance Score (FPS) (all analyses adjusted for body weight).

	**β†**	**percentage explained**	**F-value**		**β†**	**percentage explained**	**F-value**
**men**				**women**			

**FPS**		**54.2%**	24.71*	**FPS**		**36.6%**	25.70*
eye-hand coordination	.626*			reaction time	.247*		
tandem stance	-.268*			tandem stance	-.326*		
				sit and reach	-.277*		
				knee extension strength	-.161*		
**chair rise performance**		**34.2%**	21.76*	**chair rise performance**		**24.3%**	27.23*
eye-hand coordination	.598*			sit and reach	-.253*		
				tandem stance	-.214*		
**pick up a pen**		**21.5%**	11.92*	**pick up a pen**		**30.9%**	77.40*
eye-hand coordination	.484*			reaction time	.559*		
**put on a coat**		**46.2%**	12.43*	**put on a coat**		**18.6%**	20.52*
eye-hand coordination	.721*			shoulder circumduction	-.322*		
reaction time	-.436*			eye-hand coordination	.241*		
elbow extension strength	-.316*						
**walking velocity**		**44.4%**	11.67*	**walking velocity**		**33.9%**	30.20*
dorsiflexion	.314*			tandem stance	.432*		
strength ankle tandem stance	.313*			sit and reach	.290*		
eye-hand coordination	-.268*			knee extension strength	.193*		

* = significant at the .05-level, † = βvalues, standardized

The differences with respect to the predictors in the regression models confirm the necessity of gender specific analysis.

## Discussion

The objective of this study was to investigate the relationship between physical fitness components and functional performance among older people living in long-term care facilities.

The relationship between physical fitness components and functional performance differed considerably between men and women. Regression analysis revealed that in men, the main predictors of functional performance were measures for coordination, with minor contributions of measures of muscle strength. In women, on the other hand measures from all domains were predictors of functional performance, with coordination and flexibility playing a major role.

This is, to our knowledge, the first study examining the relationship between various physical fitness components and functional performance, both measured by performance-based tests, among older people living in long-term care facilities.

### Differences between male and female participants

Most previous studies examining the relationship between physical fitness components and functional performance did not discriminate between men and women. Due to the different physiological capacities of men and women, it is not surprising to find gender differences. Similar to our results, also Van Heuvelen et al. [33] found that different regression models were needed to predict function in men and women. However, they used self-reported measures to assess functional performance. Moreover, comparison of the physical fitness tests results suggests that the sample in the present study was more frail.

Samson et al. [34] investigated the correlation between knee extensor strength and functional mobility (the timed 'get-up-and-go' test, comparable to a combination of walking velocity and chair rise performance in the present study) in healthy men and women aged 19 to 90. They found stronger correlations between knee extension strength and functional mobility, both in men (r = -.60) and in women (r = -.71) than we did. Two possible explanations for this discrepancy are that first, it is not possible to translate the timed 'get-up-and-go' test into walking velocity and chair rise performance, and second, that our population was much older and more frail, and therefore not comparable to the population described by Samson et al. [34]. Other studies found only moderate correlations [13,17,35] between knee extension strength and gait speed. Brown et al. [36] pointed out that one possible explanation for the only moderate correlations found, may be that gait speed is already low in elderly, and therefore associations are less apparent. Another possible explanation relates to the differences in study populations, and the methods used for example for measuring muscle strength, which makes it difficult to compare results of different studies.

### Relationship between physical fitness and functional performance

In contrast to findings of previous studies, we did not find knee extension strength to be a major predictor of walking velocity. In men, ankle dorsiflexion strength and measures of coordination were predictors of walking velocity. In women, balance (tandem stance) and flexibility (sit and reach) were more important predictors of walking performance than knee extension strength. This indicates that walking velocity is multidimensional and that, besides knee extensor strength, also factors from other domains determine walking velocity. These findings are in concordance with the results of Brown et al. [37] who examined, among other things, the relationship between knee extension strength and overall performance, comparable to our FPS: a weak, although significant correlation was found (r = .31).

Other studies examining the relationship between walking speed and strength of the lower extremities strength, and the fact that in both studies only female subjects were measured may explain this inequality.

The results of Schenkman et al. [16] indicate that apart from lower extremity muscle strength, also balance plays an important role in functional performance of the lower extremities, for instance chair rise performance. In accordance with these findings, we found that chair rise performance could be predicted for almost one quarter by balance (tandem stance) and flexibility (sit and reach) in women. Our findings are also in concordance with the results of the study of Ringsberg et al. [17], who found walking velocity, another measure of functional performance of the lower extremities, was significantly related to balance (one leg stance) in women. Brown et al. [37] found that overall functional performance could be best explained by fitness tests measuring balance, coordination, and muscle strength. However, in their study no distinction was made between men and women.

### Limitations of the study

Buchner et al. [1] suggest that the relationship between leg strength and walking speed is non-linear. This could be an explanation for the fact that in our linear regression analysis we did not find a significant association between those two parameters. However, inspection of the correlation plots for physical fitness measures and FPS did not suggest a non-linear relationship.

Our study population did not consist of a randomly selected sample, a shortcoming that we share with many other field studies. Our study sample was recruited for participation in an exercise intervention study. This recruitment procedure will probably have favoured enrolment of a healthier and more active group of long-term care residents.

The variance in the individual functional performance scores and the total FPS explained by the individual fitness components varied between 22% and 54% for men, and between 19% and 37% for women. The remaining unexplained variance might be accounted for by other factors (e.g. medical conditions, socio-economic status) that we did not include in our analysis. Nevertheless, the aim of this study was to determine factors that are modifiable by exercise programs.

Another limitation is the lack of standardized measures physical fitness and functional performance. There is a large heterogeneity in assessment methods in studies, which makes comparison between studies difficult. Furthermore, in the present study only isometric strength tests were used and no measure of cardiovascular endurance was included. Since most subjects had difficulties with walking or used walking aids, measuring cardiovascular endurance would be difficult in our study population. Due to the large imbalance between women and men in our study population, which is common in this age category, the results and conclusions regarding the comparison of the sexes must be interpreted with care.

## Conclusion

The outcomes of this study might contribute to the development of adequate exercise programs aiming at preventing or slowing down the age-related decline of physical functioning. Coordination and to a smaller extent muscle strength seem to be important contributors to functional performance in men, while for women flexibility and coordination play a major role. These findings suggest that for improvement in functional performance, exercise programs for older men and women living in long-term care facilities should focus on different fitness components. Since coordination is an important correlate of functional performance in both, men and women, we think that in exercise programs for residents of long-term care facilities, maintenance or improvement of coordination should be emphasized.

As our results suggest that coordination and strength are important contributors to functional performance in men, one could initially argue that it is necessary to train coordination and muscle strength in men to preserve function and prevent further decline. Consistently, for women an exercise program should focus on items from the domain flexibility and coordination, as we found that these fitness measures play a major role in predicting functional performance in women.

Another possible conclusion regarding the content of exercise programs is based on the fact that, in spite of the significant different contributions of the three domains (strength, coordination and flexibility) between men and women, no significant differences were found concerning functional performance. Possibly men and women use different techniques in performing activities of daily living: men profit from their advantage in strength and they could possibly further improve their performance by increasing their flexibility. In contrast, women may benefit from strength training, making them less dependent on their advantage in flexibility. This hypothesis should be tested in an intervention study.

In conclusion, the results of this study indicate that besides muscle strength, other fitness components, e.g. coordination and flexibility, are important for functional performance of older people living in long-term care facilities. Furthermore, the gender differences concerning the factors contributing to functional performance suggest that, for men and women, exercise programs emphasizing different fitness aspects should be developed.

## Conflicts of interest declaration

The author(s) declare that they have no competing interests.

## Authors' contributions

AS, MC, RB and WvM contributed intellectual input into the main ideas of this paper. MC designed and supervised the data-collection. AS analysed the data and drafted the manuscript. MC and RB provided supervised data-analyses, interpretation of the data, and the writing. All authors contributed to the further writing of the manuscript. MC and WvM obtained financial support.

## Pre-publication history

The pre-publication history for this paper can be accessed here:


